# Harnessing YOLOv11 for Enhanced Detection of Typical Autism Spectrum Disorder Behaviors Through Body Movements

**DOI:** 10.3390/diagnostics15141786

**Published:** 2025-07-15

**Authors:** Ayman Noor, Hanan Almukhalfi, Arthur Souza, Talal H. Noor

**Affiliations:** 1 Department of Computer Science, College of Computer Science and Engineering, Taibah University, Madinah 42353, Saudi Arabia; anoor@taibahu.edu.sa (A.N.); hmukhalfi@taibahu.edu.sa (H.A.); 2 King Salman Center for Disability Research, Riyadh 11614, Saudi Arabia; arthurecassio@ppgsc.ufrn.br; 3 Department of Computation and Technology, Federal University of Rio Grande do Norte, Caicó 59078-970, Brazil

**Keywords:** Autism Spectrum Disorder, detection, deep learning, YOLOv11, body movement analysis

## Abstract

**Background/Objectives**: Repetitive behaviors such as hand flapping, body rocking, and head shaking characterize Autism Spectrum Disorder (ASD) while functioning as early signs of neurodevelopmental variations. Traditional diagnostic procedures require extensive manual observation, which takes significant time, produces subjective results, and remains unavailable to many regions. The research introduces a real-time system for the detection of ASD-typical behaviors by analyzing body movements through the You Only Look Once (YOLOv11) deep learning model. **Methods**: The system’s multi-layered design integrates monitoring, network, cloud, and typical ASD behavior detection layers to facilitate real-time video acquisition, wireless data transfer, and cloud analysis along with ASD-typical behavior classification. We gathered and annotated our own dataset comprising 72 videos, yielding a total of 13,640 images representing four behavior classes that include hand flapping, body rocking, head shaking, and non_autistic. **Results**: YOLOv11 demonstrates superior performance compared to baseline models like the sub-sampling (CNN) (MobileNet-SSD) and Long Short-Term Memory (LSTM) by achieving 99% accuracy along with 96% precision and 97% in recall and the F1-score. **Conclusions**: The results indicate that our system provides a scalable solution for real-time ASD screening, which might help clinicians, educators, and caregivers with early intervention, as well as ongoing behavioral monitoring.

## 1. Introduction

The distinctive behavioral patterns exhibited by children with Autism Spectrum Disorder (ASD), including hand flapping, body rocking, and head shaking, reveal early neurodevelopmental differences [1,2]. The early detection of these behaviors enables prompt diagnosis and intervention, which yields significant positive results in communication development as well as learning and social skills improvement. The traditional methods for diagnosing ASD require subjective evaluations, which are time-consuming and rely on expert observations conducted in controlled settings [3,4,5]. The limitations currently present in clinical assessment procedures extend waiting times and limit early intervention program accessibility, especially in regions lacking resources. Current diagnostic challenges for ASD increase the need for automated systems that can operate in real-world settings to accurately detect ASD behaviors and reduce diagnostic bias.

Conventional ASD screening methods depend on structured behavioral checklists together with caregiver interviews and manual video review, which introduce observer variability and limited temporal resolution [6,7]. These methods have proven essential for developmental evaluations, but they often fail to detect detailed, repetitive behaviors that occur in unstructured settings. Deep learning advancements demonstrate potential for facial expression and speech signal analysis and physiological indicators [8,9], yet full-body movement patterns remain underutilized in behavioral detection studies. The potential benefits of motor-based cues have led researchers to start investigating video-based ASD detection systems, which currently face limitations in real-time processing capabilities as well as robustness to background disturbances and the detection of subtle movements. The existing detection challenges in ASD monitoring necessitate a responsive detection framework that integrates multiple elements to meet real-world monitoring requirements.

Advances in computer vision and deep learning techniques have transformed behavioral analysis through object detection models that enable real-time inference. The You Only Look Once (YOLO) family emerges as a top architecture because of its capability to quickly and precisely detect dynamic behaviors across spatial and temporal dimensions [10,11]. YOLOv11 utilizes lightweight modules, including EfficientRepNet, SPPF, and C2PSA, which enhance its performance in detecting motion-based cues within video frames while maintaining a high processing speed. YOLOv11 demonstrates strong performance for analyzing ASD-related movement patterns in noisy video feeds. YOLOv11 enables clinicians, caregivers, and educators to perform continuous behavior monitoring and early screening through real-time detection of hand flapping and head shaking behaviors while helping to create diagnostic systems that are both objective and scalable.

While previous research has focused on facial expressions, EEG signals, and audio signals, this research work uniquely applies real-time object detection to body movement analysis for ASD-typical behavior classification. Our system takes advantage of YOLOv11 technology in a modular cloud architecture to deliver scalable behavior monitoring solutions with low-latency features. The development of an expert-validated dataset combined with empirical validation tests on multiple model baselines demonstrates the technical and practical innovation of our research.

The presented paper introduces a new ASD-typical behavior detection system that utilizes YOLOv11-based deep learning to analyze body movements. The system operates through a modular framework to achieve low latency and high performance when it captures repetitive ASD behaviors from video frames for classification. Our system architecture includes four core components, including the real-time optimization of the *monitoring*, *network*, *cloud*, and *ASD-typical behavior detection* layers, enabling effective data collection, transmission, and inference throughout diverse application settings. The key contributions are summarized as follows:

- To the best of our knowledge, this research is one of the first research works that uses YOLOv11 to identify ASD behaviors through real-time analysis of upper body movements, including hand flapping, body rocking, and head shaking.
- Our system enhances live video streams’ spatial–temporal behavior recognition by integrating the YOLOv11 object detection model with customized modules such as C3k2, SPPF, and C2PSA.
- We have gathered our own dataset comprising 72 videos, yielding a total of 13,640 images, which consist of four different behavior categories, which have been collected and annotated from public sources with validation by certified autism specialists.
- Our benchmarking results show that YOLOv11 outperforms both CNN (MobileNet-SSD) and LSTM models in ASD behavior detection through improved accuracy, precision, recall, and F1-score metrics.

The structure of the following sections of this paper is detailed as follows: Section 2 provides an overview of existing ASD-typical behavior detection methods that utilize deep learning coupled with computer vision techniques. Section 3 introduces the system architecture for autism detection based on body movements. Section 4 introduces the YOLOv11 model alongside its behavior classification components. Section 5 details the implementation and experimental setup. Section 6 provides the evaluation results and performance comparisons. Section 7 concludes the paper while presenting future work directions.

## 2. Related Work

The latest Autism Spectrum Disorder (ASD) detection studies have utilized multiple data modalities alongside various machine learning (ML) techniques to pinpoint behavioral, physiological, and cognitive markers of the disorder. To provide a clearer comparative overview, we organize this section based on the primary type of data used. Detection approaches for ASD include (i) image-based systems utilizing facial and neuroimaging data; (ii) video-based methods that examine motion and behavior patterns; (iii) vocal pattern analysis through speech- and audio-based technologies; and (iv) clinical screening tools supported by questionnaire and metadata-driven techniques. Our comparison involves distinguishing traditional ML models from deep learning (DL) models in each data category and explaining the data origins and research conditions when available.

### 2.1. Image-Based ASD Detection Approaches

Mujeeb et al. [12] compare deep learning models for diagnosing Autism Spectrum Disorder (ASD) in children based on static facial expressions. They trained sub-samplings (CNNs) such as MobileNet, Xception, and EfficientNet using transfer learning on the faces. ASD-related facial features (i.e., a wider upper face and wider eyes) were used to separate autistic from normal children. It used 2936 faces from 2- to 14-year-olds, which were equally split into the ASD and non-ASD populations. The most robust model was Xception with an AUC of 96.63%, which is suggestive of deep learning models as non-invasive ASD screening tools. Yet the research admits that data is somewhat scant and calls for further investigation before clinical trials.

Beary et al. [13] propose a face image analysis approach to define autistic and non-autistic children. The authors used a CNN modeled on MobileNet and obtained a diagnostic accuracy of 94.64% for autism using unique facial characteristics, including a broad upper face and a shorter middle part of the face. There were 3014 images in the dataset (i.e., across autistic and non-autistic children equally), although some doubts about dataset quality and validation were expressed. This work suggests that facial analysis can be a fast and affordable way to screen for autism early, and improvements are planned for better outcomes.

Awaji et al. [14] focus on helping the early detection of ASD using CNNs as the basis model and machine learning approaches. The models in the experiment were the CNNs VGG16, ResNet101, and MobileNet, as well as a combination of XGBoost and RF classifiers. Dimensionality embedding was carried out with T-distributed Stochastic Neighbor Embedding (T-SNE). These images included two thousand nine hundred and forty children’s faces taken from Kaggle and were preprocessed to crop the images and normalize them. VGG16 + XGBoost came out with 95.7% and MobileNet + the Random Forest with 95.9%. In this way, CNN feature-based and primitive machine learning hybrid models were superior in terms of precision, sensitivity, and specificity. It was a study that focused on early detection in children with a focus on its clinical future.

Farhat et al. [15] focus on establishing a fast and affordable diagnostic system for ASD based on facial images, particularly for under-resourced diagnostic areas such as Pakistan. In the study, VGG16 and MobileNet deep neural networks were trained to pick up autism instead of base images. The method uses CNNs for image feature extraction and 5-fold cross-validation to ensure robustness. The dataset was retrieved from Kaggle and included 2500 training, 100 validation, and 300 testing images of autistic and non-autistic children (i.e., ages 2–14 years). To extend it to the field, 50 additional images were taken. With 99% validation and 87% testing accuracy, the VGG16 classifier was the top-performing one, with MobileNet being one notch lower. Executing the model locally yielded 85% accuracy (i.e., indicating its usefulness for ASD detection). This research demonstrates the potential of machine learning to enhance the diagnosis of autism in areas with restricted diagnostic tools.

Sharma and Tanwar [16] present a CNN-based diagnosis of ASD using neuroimaging data. The authors trained their model on the Autism Brain Imaging Data Exchange (ABIDE) dataset (i.e., brain scans of autistic and non-autistic people). They leveraged deep learning to obtain 93.41% accuracy, showing that the model can pick up ASD based on patterns of brain connectivity. It stresses early detection as the key to a better life for people with ASD, and the model is superior to more traditional machine learning methods. Thanks to good preprocessing and the brain areas analyzed, this study could be used for automatic ASD diagnosis.

Ahmad et al. [17] examine whether any of the pre-trained CNNs can detect ASD on the basis of face recognition images. Images in this study were drawn from Kaggle datasets labeled for ASD detection based on visual traits. The findings also point to the critical role of early detection of ASD in ensuring enhanced development for those with ASD. Through transfer learning, the model comparison of ResNet34, ResNet50, AlexNet, MobileNetV2, VGG16, and VGG19 showed that the ResNet50 model had the highest accuracy at 92%. It also addresses the data used (i.e., images of autistic and non-autistic children), the DL algorithms used, and the issues and limitations of diagnostic tools for diagnosing ASD today.

Jiang et al. [18] solve the problem of how to accurately classify individuals with ASD while performing facial emotion recognition tasks by comparing eye movements and task performance between ASD participants and typical development (TD) controls. The idea is that the combination of eye tracking and facial recognition improves classification accuracy when it comes to ASD individuals. The proposed model is based on a Random Forest (RF) classifier that takes task performance, gaze, and high-density facial features generated with the OpenFace deep neural network. The model was trained on a set of 23 ASD subjects and 35 TD controls, who had to perform a facial emotion recognition task while their eyes were tracked. The outcome was that the combined model performed well, achieving an overall accuracy of 86% and a sensitivity of 91.3%, highlighting the importance of eye-tracking data in separating ASD individuals. It also points out how machine learning can be used to provide objective diagnostic ASD assessments by harnessing behavioral and eye-tracking data for improved classification.

### 2.2. Video-Based and Body Movement Detection Approaches

Alcaniz et al. [19] use virtual reality (VR) and machine learning (ML) to categorize the body movements of autistic children. The project sought to develop a quantitative approach to better diagnose ASD, which is normally qualitatively assessed. In the study, 24 ASD and 25 children with TD participated. The dataset used consisted of body movement recordings collected via depth-sensor cameras in a controlled VR environment, which was specifically designed for ASD evaluation, with annotated behaviors captured during imitation tasks. The children were immersed in VR environments, performing imitation tasks with their body movements tracked by depth-sensor cameras. The researchers discovered that ASD children had larger and more frequent movements, particularly in the head, trunk, and feet, than TD children. The VR system used a range of stimuli (i.e., viz, visual, auditory, and smell) to monitor children’s responses under different conditions. The most significant results indicated that head movements, especially those following visual stimuli, were the most characteristic for ASD (i.e., with classification success at 82.98%). The machine learning approach enhanced ASD classification, with the highest sensitivity reaching 89.36% using visual stimuli. The researchers conclude that VR paired with machine learning is a promising, goal-directed method for identifying ASD through body movement. This approach could complement conventional diagnostic procedures by ensuring greater ecological credibility and less subjective judgment.

Prakash et al. [20] focus on the issue of evaluating children with ASD using manual, time-consuming, and inconsistent methods. Computer vision and deep learning can be automated and can improve the accuracy of analyzing ASD children’s interactions, emotions, and skills so that they can be completed quickly and more reliably. The authors outline three deep learning (DL) models, which are activity comprehension (AC) for assessing child–play-partner relationships, joint attention recognition (JAR) with head and hand pose tracking for joint attention skills, and facial expression recognition (FER) for emotion detection in video recordings. The JAR eye gaze tracking model produced the highest rate at 97%. It has 300 videos of ASD children participating in play interventions and 68 actual test videos. These models support clinicians in automating the extraction of behaviors, emotions, and social abilities so that ASD children can be better diagnosed and treated.

Tariq et al. [21] focus on delayed autism diagnoses that are caused by the manual, long-awaited nature of behavioral testing and subsequent waiting lists for therapy. This hypothesis proposes that machine learning-based home video analysis is faster and more precise than clinical diagnosis. The proposed technique includes mining 30 behavioral characteristics from short home videos and mulling through eight ML models. The most efficient model is the five-feature Logistic Regression (LR5) classifier, which returns 88.9% accuracy, 94.5% sensitivity, and 77.4% specificity. This model was tested on a separate set of 66 videos, yielding an accuracy of 89% (i.e., which is slightly less). The data includes 162 videos, of which 119 are of autistic children and 46 are of normally developing children. The researchers conclude that this mobile video analysis is a scalable and effective tool for early autism detection, potentially breaking down diagnostic bottlenecks and improving access to intervention in real time.

### 2.3. Speech- and Audio-Based ASD Detection Approaches

Lee et al. [22] focus on speech patterns in autism by machine learning. It also parses speech information from ASD patients using audio files from the ADOS. Their study uses machine learning algorithms (i.e., Gradient Boosting) to sort ASD speech characteristics (i.e., intonation, volume, and rhythm). The dataset is speech samples that have been extracted and analyzed with prosodic analysis programs. The Gradient Boosting model had an accurate of 87.75%, which makes it a promising model for ASD diagnosis. However, the paper also notes drawbacks such as the data size and cross-language testing to generalize the model.

Ramesh and Assaf [23] explore ML approaches for the diagnosis of ASD using speech information. Early ASD diagnosis is essential for a good intervention, but current practices are slow and subject-focused. Their work is focused on the computational linguistics and machine learning analysis of the speech of ASD children and TD children. The analysis is based on TalkBank, an enormous spoken language dataset, and includes five ML classifiers (i.e., LR, RF, SVM, NB, and KNN). We sifted through the speech data to find features like the mean length of utterance and parts of the speech that were put into the models. The Logistic Regression and Random Forest models were the best at predicting ASD with 75% accuracy. Overall, the research finds that there is room for improvement, but ML models provide a robust, non-invasive tool to diagnose ASD from speech data. Future work will be focused on improving the model and scaling the app across age groups.

Hu et al. [24] analyze speech deficits in people with ASD using machine learning. Using recordings of Autism Diagnostic Observation Schedule (ADOS) sessions, the team strips out speech patterns, including intonation, rhythm, and volume, in order to distinguish ASD patterns. Gradient Boosting and other machine learning models were extremely accurate with 87.75% accuracy. The paper also highlights the promise of speech-based diagnosis in early ASD diagnosis but suggests that it requires additional testing across populations and languages to make it more generalizable.

Ali et al. [25] employ speech traits from voice recordings to distinguish children with ASD from normal children through Electroenc-Ephalo-Graphic (EEG) signals. The authors used the EEG of 20 participants (12 normal and 8 ASD) aged 9–16 and used a six-layer CNN to categorize the brain waves. The classification accuracy was around 80%; the small dataset and difficulties in interpreting the spatial correspondences within EEG signals hinder the generalization of the model. The paper notes that EEG analysis offers promising results for ASD detection, but it does not disregard larger datasets and better model architectures.

Farooq et al. [26] are concentrating on the privacy-preserving use of federated learning (FL) to diagnose ASD. FL supports the training of machine learning models locally, without relocating sensitive data to a central server. Two classifiers, Logistic Regression (LR) and the Support Vector Machine (SVM), were trained on four datasets consisting of speech and non-speech communication, habitual behaviors, and sensory perception. The highest accuracy was achieved with the SVM, 99.0% for children and 81.0% for adults. Its results show that FL can provide effective and privacy-preserving ASD detection across age groups more effectively than traditional ML algorithms and call for future work in image-based diagnosis and transfer learning for prediction enhancement.

### 2.4. Questionnaire-, Sensor-, or Metadata-Based Approaches

Sujatha et al. [27] analyze whether ML can be used to identify patients with ASD according to their family history and responses to questionnaires that diagnose the disorder. There were seven different algorithms used: Support Vector Machines, K-Nearest Neighbors (KNNs), RF, Naive Bayes (NB), Stochastic Gradient Descent (SGD), AdaBoost, and CN2 Rule Induction. It was the dataset from the University of California Irvine (UCI) ML repository, which was further subdivided into toddlers, children, adolescents, and adults. The most accurate models (i.e., with respect to the performance indices) differed by age groups: for toddlers, AdaBoost achieved 99.8% accuracy and 97.2% for adolescents, while SGD achieved 99.6% accuracy and 99.7% accuracy for children and adults, respectively. Each algorithm performed well across most age ranges based on data complexity as well as age group. These results suggest that some of the ML models based on the Random Forest/SGD are accurate in defining ASD patients and need more experiments based on deep learning parameters.

Rasul et al. [28] used a variety of ML methods, such as the SVM and LR, to identify ASD early. The study utilizes tabular questionnaire responses and metadata from ASD and non-ASD individuals in both adult and child populations, with an accuracy of 99.14% for children and 99.143% accuracy for adults. Their study studies feature selection and hyperparameter optimization to obtain a better diagnostic performance. But it is limited by a lack of comprehensive clinical data and real labels to perform supervised learning.

Jayaprakash and Kanimozhiselvi [29] examine ML approaches to identify children with a high risk of ASD. Using the Childhood Autism Rating Scale (CARS) behavioral assessment, they ran different models of multinomial Logistic Regression across children 6 months to 5 years of age. The analysis showed decent accuracy, with the Newton-CG solver at 97%. The results will hopefully help clinicians diagnose ASD in time, albeit with limitations on a larger dataset and potential behavioral biases.

Akter et al. [30] combine the use of various machine learning algorithms (i.e., SVM, Adaboost, and Glmboost) on metadata and screening questionnaire responses from both UCI and Kaggle sources to detect ASD in early phases and in all age groups. The highest accuracy rates, 100%, were achieved for age datasets, where Adaboost performed the best for children and adults. However, the study had a limited dataset and needed more data to determine the risk factors.

Hossain et al. [31] investigate ML methods to detect ASD. The authors analyzed 25 classification techniques on datasets (i.e., toddler, child, adolescent, and adult), and the SVM using sequential minimal optimization (SMO) was the most accurate and efficient. For feature selection, such as information gain and relief to pick the most important features, screening responses outweighed demographics. The SMO-based SVM obtained 100% of the correct answers, demonstrating that machine learning can help perform ASD diagnosis easier. The authors recommend the use of deep learning methods in the future for even better detection.

Erkan and Thanh [32] consider ML-based diagnosis of ASD. They experiment on algorithms such as the SVM, LR, and CNN to model children, adolescents, and adults datasets. The results indicate that CNN-based models outperform other models with a 99.53% accuracy rate in child ASD detection. The article highlights how machine learning can be used to help diagnose ASD earlier and accurately so that treatment can begin as early as possible and improve outcomes for those with ASD.

Although the reviewed literature provides strong advances in applying DL and ML models to a wide range of ASD data (i.e., facial images, EEG data, and behavior), our study stands apart from the rest by applying the YOLOv11 DL model to analyze body movements in ASD patients. In contrast to earlier work that was generally concerned with wider-ranged behavioral analysis or facial recognition, our system specifically focuses on the detection of body motor variations. Our approach promises to reveal a richer understanding of body motor signals as potential markers for ASD, bringing advanced methods of motion analysis together. This would have a dramatic impact on early ASD diagnosis and, as a result, on outcomes in subsequent interventions. Our work not only fills a crucial gap but also innovates how DL can be leveraged within the healthcare technology industry to enhance diagnostic and therapeutic results.

## 3. Body Movement-Based Autism Spectrum Disorder Detection System Architecture

**Figure 1:**
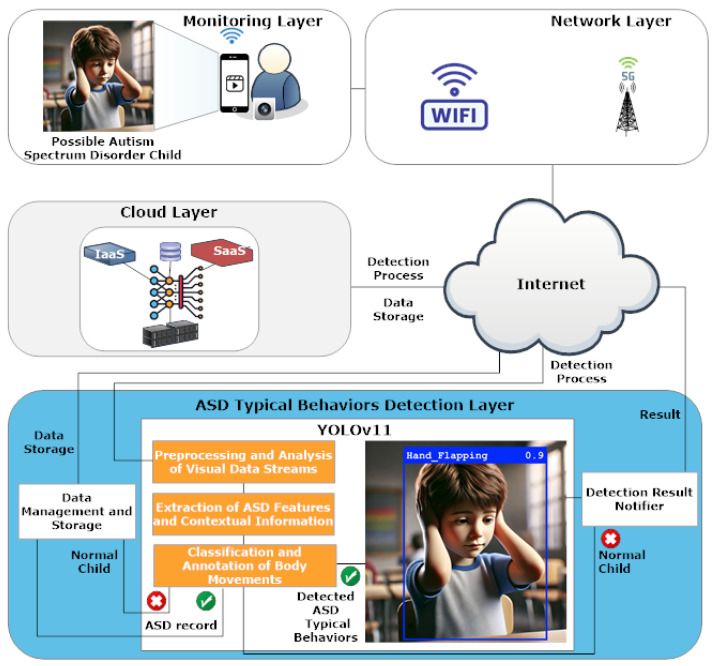
Body Movements-based ASD-typical behavior detection system architecture.

The body movement-based Autism Spectrum Disorder (ASD)-typical behavior detection system, designed to monitor body movements in children, uses a multi-layered architecture that combines behavioral monitoring with wireless communication and cloud computing to apply deep learning for ASD-typical behavior detection. The system architecture analyzes body movements in real time and accurately classifies behavior through the YOLOv11 model. The system’s architecture consists of four main layers, including *monitoring*, *network*, *cloud*, and *ASD-typical behavior detection*, which perform tasks that cover data acquisition to behavioral classification and user notification as detailed in Figure 1. The layered system structure supports uninterrupted monitoring and quick data transfer while protecting data storage and enabling advanced ASD-typical behavior detection, which promotes early diagnosis alongside intervention support.

The detection pipeline depends on the essential functions of every system layer. The layers are described below:

1. *Monitoring Layer:* The monitoring layer captures the raw visual information of the children using closed-circuit television (CCTV) or mobile devices, such as a phone or a tablet, in supervised and consented environments like clinics, homes, or research laboratories, which can be used to monitor children for whom monitoring has been requested. The goal of the system is to provide supportive information that can identify any repetitive body movements that could indicate the presence of typical ASD-related behaviors, but it should not be used to diagnose a child with ASD. It is not designed to operate in public spaces or crowded places like schools or playgrounds, where ethical and privacy considerations require strict regulatory frameworks beyond the scope of this system. Visual data collection is sent to the *network Layer* where processing continues.
2. *Network Layer:* The network layer enables wireless links that connect monitoring devices with cloud computing systems. The system uses Wi-Fi and 5G technologies to support fast video streaming and sensor data transmission while reducing latency. The network enables the immediate transmission of captured behavior data, which can then be analyzed.
3. *Cloud Layer:* The cloud layer provides scalable resources for computation and storage via Infrastructure as a Service (IaaS) and Software as a Service (SaaS). The system securely stores visual data that arrives while also running behavioral classification models. Through its modular structure, cloud services provide dynamic processing capabilities and centralized behavioral data management for streamlined model deployment and expanded data scalability.
4. *ASD-Typical Behavior Detection Layer:* This layer performs preprocessing of visual data, extraction of children’s ASD-typical behavioral features, and classification of ASD children’s behavior and annotation. The core layer executes the YOLOv11 deep learning model while supporting multiple functional modules. The YOLOv11 model leverages the following essential components:
  - Preprocessing and Analysis of Visual Data Streams: Several preprocessing steps are executed by the system to make video frames ready for classification. The preprocessing steps involve resizing video frames to 640 × 640 pixels to fit YOLOv11 input requirements, normalizing pixel intensity values, and converting ground-truth annotations to YOLO labels. The data receives augmentation treatment by applying random horizontal flipping, brightness adjustments, and cropping so that the model can recognize various lighting conditions along with different camera angles and subject positions typical of real-world scenarios.
  - Extraction of ASD Features and Contextual Information: The EfficientRepNet backbone initiates feature extraction by processing each input frame through multiple convolutional layers that capture hierarchical spatial representations. Edge pattern and fine texture feature extraction happens at low-level layers, while deeper layers detect mid- and high-level motion features that match repetitive body movements like hand flapping and head shaking. The Spatial Pyramid Pooling Fast (SPPF) module improves feature extraction by combining contextual data from various receptive fields, while the Cross-Stage Partial with Parallel Spatial Attention (C2PSA) block focuses detection efforts on spatial areas that display rhythmic or cyclical movement patterns. These modules work in unison to allow the system to identify important movement dynamics for ASD-typical behavior detection even in complex or noisy surroundings (e.g., hand flapping, body rocking, and head shaking). Further details on ASD feature extraction can be found in Section 4.
  - Classification and Annotation of Body Movements: The YOLOv11 model performs behavioral classification while it attaches labels and confidence scores to identified actions. It identifies ASD-indicative actions in real time.

Other modules are used for data storage and notification for caregivers or health professionals. The modules include

- Data Management and Storage: The system stores behavior annotations together with essential metadata, which includes timestamps along with labels and detection scores. This facilitates longitudinal analysis and historical tracking.
- Detection Result Notifier: The system provides detection outcomes to caregivers or clinical systems, which indicate whether the observed behavior falls into typical or ASD-related categories. The module generates practical information to enable immediate follow-up actions.

It is important to note that the system functions to identify ASD’s typical behavioral features but does not directly diagnose ASD. The system recognizes specific behavioral signs associated with ASD, including hand flapping, body rocking, and head shaking, which are confirmed markers in clinical and diagnostic research [1,2]. The non_autistic class models normal behavioral patterns to distinguish everyday movements from ASD-linked activities. A fifth class called background contains frames without subjects or irrelevant activity, which helps decrease false positives while training models. The identification of patterns associated with ASD functions as a preliminary alert tool that aids caregivers and clinicians by indicating early motor signs that require additional evaluation.

## 4. YOLOv11 Model for ASD-Typical Behavioral Classification

The YOLOv11 model forms the foundation of the body movement-based Autism Spectrum Disorder (ASD)-typical behavior detection system, which detects and classifies behavioral patterns that indicate ASD. The You Only Look Once (YOLO) family of models is well-known through its combination of deep learning-based object detection technology and real-time inference functionality [33]. YOLO has progressed over its versions to improve detection accuracy and efficiency through the enhancement of its architecture and training methods [10,11,34,35,36]. YOLOv11 progresses the existing advancements through the inclusion of specially optimized modules that enable real-time classification of brief behavioral patterns, which are crucial for the detection of ASD [37].

YOLOv11 uses EfficientRepNet as its backbone to extract deep features efficiently while maintaining a low computational load, which is important for analyzing dynamic movements in live video frames [38]. The model features a transformer-based lightweight neck module that enhances contextual learning while enabling dynamic label assignment to better distinguish overlapping or similar behavior sequences. The system architecture maintains YOLO’s detection grid design, which enables each cell in an S×S grid to generate bounding boxes along with class probabilities for detected behaviors located at the center of each cell.

YOLOv11 was selected for the ASD-typical behavior detection system because it satisfies essential system requirements. YOLOv11 meets system demands with its rapid processing speed combined with precise detection capabilities while maintaining dependable operation in resource-constrained environments. Detecting ASD movement indicators such as hand flapping, body rocking, and head shaking behaviors (i.e., demands a model that can accurately track fast upper-body movements with both spatial and temporal precision [39,40,41,42].

The system architecture uses three specialized modules that improve detection accuracy and spatial awareness during behavior classification tasks.

- **C3k2 Block**: The Cross-Stage Partial with Kernel Size 2 (C3k2) block transforms conventional CSP bottlenecks to boost efficiency through the replacement of large convolutions with sequential pairs of smaller ones. The C3k2 block improves the extraction of basic motion patterns that enable the precise detection of small-scale movements like hand flapping. It is defined in Equation (1):
  (1) C3k2(ξ)=ϕ(η(ξ))+ϕ(τ(η(ξ)))
  where ξ denotes the input feature map, η(·) splits it into two branches, ϕ(·) represents a convolution operation, and τ(·) merges the bypassed and processed paths using a final convolution layer.
- **SPPF Block**: The Spatial Pyramid Pooling Fast (SPPF) block enlarges the model’s receptive field through multiple pooling layers, which merge both local and global context features. The model gains enhanced capability to identify repetitive motion patterns over different time durations, as illustrated with continuous body rocking movements. The module is represented in Equation (2):
  (2) SPPF(ξ)=κ(π(ξ,5),π(ξ,3),π(ξ,1))
  The operator π(ξ,k) performs max pooling using kernel size *k* while κ(·) merges these feature maps along the channel dimension.
- **C2PSA Block**: The Cross-Stage Partial with Parallel Spatial Attention (C2PSA) block enhances spatial attention by directing focus to areas of movement activity within video frames, such as persistent head tilting. Focusing attention on key areas inside the frame lowers false positive rates and boosts the detection of behaviors obscured from view or displaying weak signals. The block’s function is captured in Equation (3):
  (3) $$C2\mathrm{PSA}\left(\xi \right)=\gamma (\kappa \left(\xi_{\alpha },\xi_{\beta }\right))$$
  The input feature map ξ undergoes parallel processing which creates the branches $\xi_{\alpha }$ and $\xi_{\beta }$. The outputs undergo concatenation before being refined through the spatial attention function γ(·) that highlights crucial behavioral signals.

The integration of multiple modules allows YOLOv11 to accurately identify ASD-related behaviors during live video while maintaining efficient performance. The system’s modular architecture enables it to adapt across various ASD-typical behavior detection applications and video settings. The model combines fast processing with strong contextual understanding to identify slight body movements linked to neurodevelopmental symptoms in children, which enables prompt behavioral evaluations and clinical follow-up.

Traditional video action recognition models such as I3D and C3D analyze complete video sequences to identify activities, yet our study adopts an object detection technique through YOLOv11 for multiple important reasons. The ASD behaviors we focus on (hand flapping, body rocking, and head shaking) involve brief repetitive movements that take place in specific areas of video frames. Instance-level behavior patterns that are spatially confined perform better in detection through object detectors as compared to sequence-based models, which focus on wider temporal dynamics. YOLOv11 achieves fast inference speeds with both low latency and high accuracy, which proves crucial for live use cases within clinical and educational environments. Object detectors generate bounding box outputs, which enhance system interpretability by allowing caregivers to inspect and analyze the specific areas where ASD-relevant behaviors are detected. The benefits of object detection make it a superior and practical option for behavioral recognition than conventional action recognition techniques.

## 5. Implementation and Experimental Setup

Our ASD-typical behavior detection system, based on body movements, used PyTorch (https://pytorch.org/) (v2.2.2) and OpenCV (https://opencv.org/) for video processing and dataset preparation. For evaluation purposes, YOLOv11 was implemented using the Ultralytics framework (https://docs.ultralytics.com/models/yolo11/, accessed on 12 February 2025). In addition, we implemented a sub-sampling CNN (MobileNet-SSD) with the TorchVision detection API. For the Long Short-Term Memory (LSTM) model, we used the PyTorch-based framework to process sequential video frame features extracted through a pre-trained CNN backbone. The NVIDIA Tesla V100 GPU (**Equipment source**: NVIDIA Corporation, 2788 San Tomas Expressway, Santa Clara, CA, USA (GPU core fabricated by Samsung, South Korea)), (32 GB of VRAM and 5120 CUDA cores) served as the hardware platform for training and evaluating all models to maintain consistent benchmarking results.

### 5.1. Dataset Description

Our ASD-typical behavior detection system received training and evaluation through a custom dataset that we gathered from publicly available videos sourced from YouTube and educational archives via web crawling. The raw videos were filtered and annotated to extract behavior-specific content representing four target classes: the classes extracted from the raw videos include hand flapping, body rocking, head shaking, and non_autistic.

A total of 3410 manually verified frames make up each class, producing a balanced dataset with 13,640 annotated images (i.e., produced from 72 videos), all reviewed and confirmed by certified autism specialists from the Shuaa Al-Amal Center (https://www.shuaa-alamal.com/care, accessed on 17 March 2025). In order to reduce sensitivity to background structures, illumination, and viewpoints, the data were augmented by horizontal flipping, brightness adjustment, and random cropping. The detection models achieved consistent input dimensions by resizing all frames to 640 × 640 pixels. To prevent data leakage and preserve the integrity of the validation process, we applied a video-level split strategy, where frames extracted from each video were designated solely to the training set or validation set without overlap between the two. The dataset division resulted in 80% for training purposes, while 20% was reserved for validation. During training, the model underwent data augmentation through horizontal flipping and brightness variation, together with random cropping to enhance generalization and strengthen resistance to real-world movement and lighting changes. Furthermore, we performed 5-fold cross-validation to improve performance, generalization, and measure uncertainty. The dataset of 72 videos underwent a random division into five separate folds, with each fold containing unique video sources to maintain complete separation. During every round of validation, one fold served as validation data while the other four folds served as training data. The configuration ensures complete separation between the training and validation frames, which enables thorough model assessment (i.e., the video-wise split was such that the same video environment was not included in both the training and validation sets).

**Figure 2:**
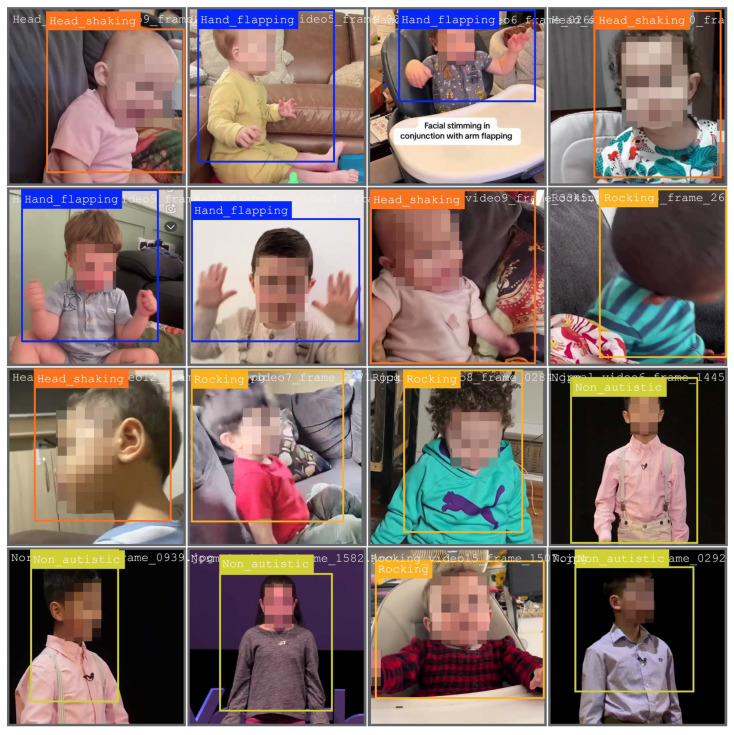
Sample validation batch annotations used with YOLOv11 for the hand flapping class.

**Figure 3:**
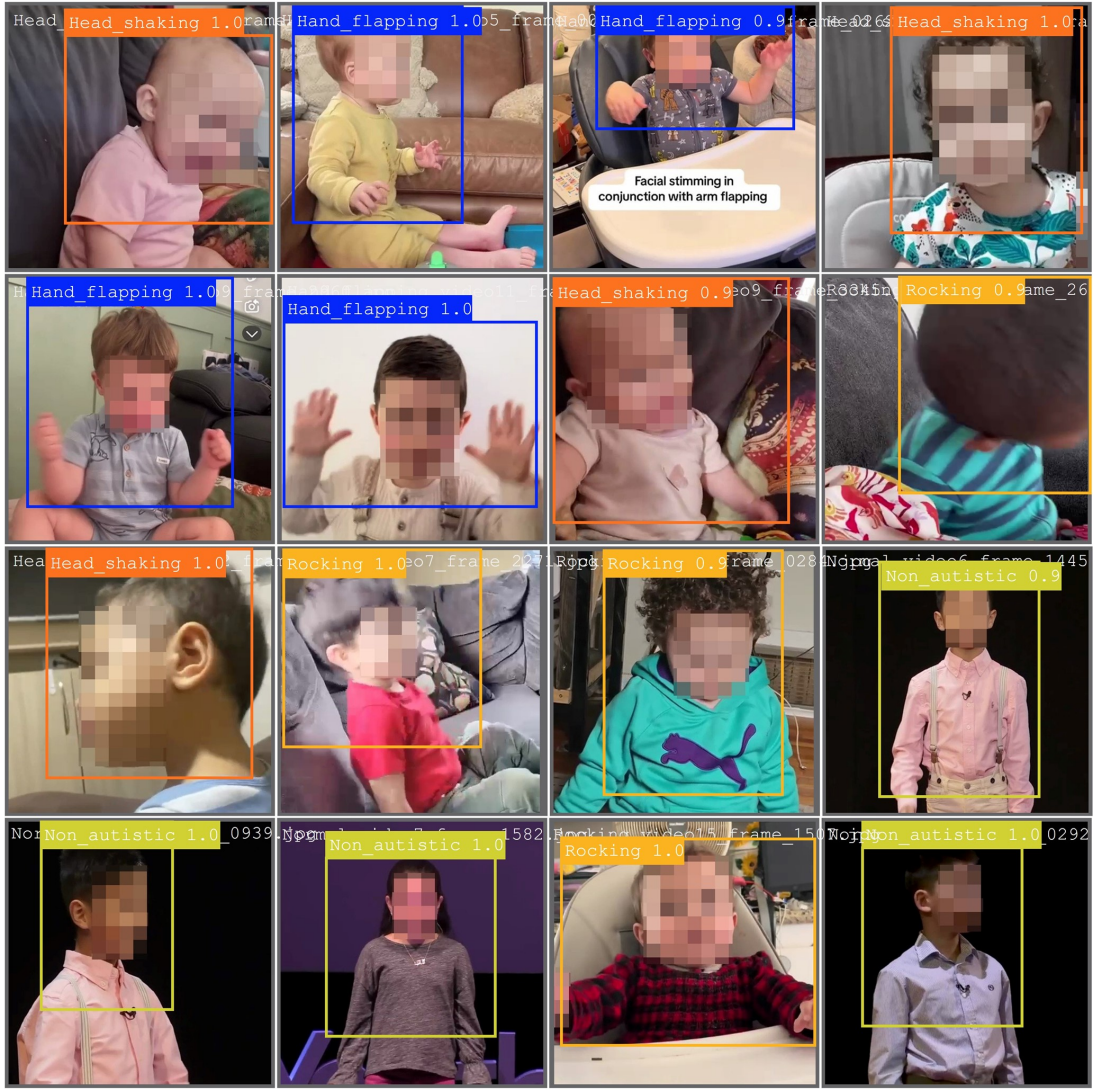
Bounding box predictions from YOLOv11 on the same validation batch for the hand flapping class.

Figure 2 displays annotated frames utilized for training purposes, while Figure 3 presents the bounding box predictions made by YOLOv11 in the validation phase.

### 5.2. Training Configuration and Hyperparameters

The training process for all models was carried out separately with settings specific to their behavior requirements. YOLOv11 achieved real-time detection of detailed movement signals, while MobileNet-SSD functioned as a compact CNN standard, and the LSTM model assessed behavior patterns across time periods drawn from moving frame sets. Table 1 shows the training hyperparameter settings for all models.

We chose hyperparameters by integrating preset values from earlier studies with hands-on experimental adjustment. We used Ultralytics-recommended [43] configurations as our initial setup for YOLOv11 and refined them by conducting a limited grid search across learning rates (0.001–0.01), batch sizes (16–64), and optimizer types (SGD vs. Adam). The validation split performance determined the final parameter settings. The optimal learning rates and batch sizes for MobileNet-SSD and LSTM models were identified through manual adjustment based on validation loss and accuracy patterns during the first 10 training epochs. Resource constraints prevented a complete hyperparameter sweep, but our selected values allowed fair convergence and stable training for every model variant.

YOLOv11 used automatic anchor box generation while mixed-precision training helped it achieve better memory efficiency and faster convergence times. The LSTM model processed CNN feature embeddings from sequential frames to track temporal patterns and movement continuity, which helped classify behaviors such as rocking and flapping.

## 6. Experimental Results

The experimental evaluation aims to determine the model’s effectiveness in detecting and classifying three repetitive behaviors (hand flapping, body rocking, and head shaking), which are commonly linked to ASD. Instead of serving as a clinical diagnosis tool, the model’s classification results should be utilized for early behavioral screening and monitoring. The system displays robust capabilities by accurately separating these behavior categories from both non_autistic and background classes, thus enabling the reliable identification of high-risk patterns that require clinical attention. The non_autistic class models normal behavioral patterns to distinguish everyday movements from ASD-linked activities. A fifth class called background contains frames without subjects or irrelevant activity, which helps decrease false positives while training models.

**Figure 4:**
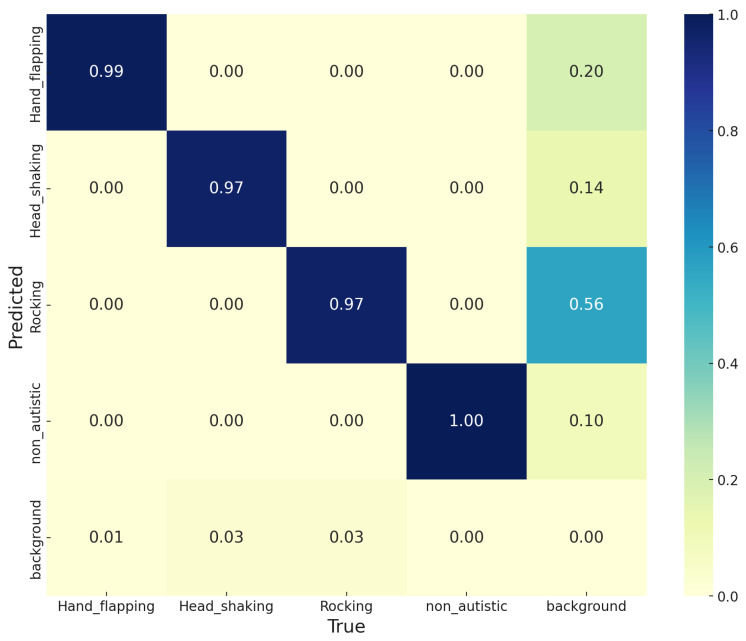
Normalized confusion matrix illustrating the classification performance of the YOLOv11 model.

Figure 4 presents the normalized confusion matrix illustrating the classification performance of the YOLOv11 model across five categories, including *Hand_flapping*, *Head_shaking*, *Rocking*, *non_autistic*, and *background*. The YOLOv11 model achieves 99% accuracy in identifying *Hand_flapping*, 97% accuracy for both *Head_shaking* and *Rocking*, and an accuracy of 100% when detecting *non_autistic* frames. The matrix indicates that the *background* class faces misclassification difficulties with a 56% confusion rate for the *Rocking* category, which shows that many background frames were wrongly identified as active ASD behavior (i.e., due to the rocking activity). The prediction of *background* resulted in fewer misclassifications when mistaken for *Hand_flapping* and *Head_shaking*, scoring 1% and 3%, respectively. The findings not only demonstrate YOLOv11’s proficiency in detecting behavior but also show necessary improvements for accurately distinguishing between movement-based and static visual inputs.

### 6.1. YOLOv11 Model Performance Metrics

**Figure 5:**
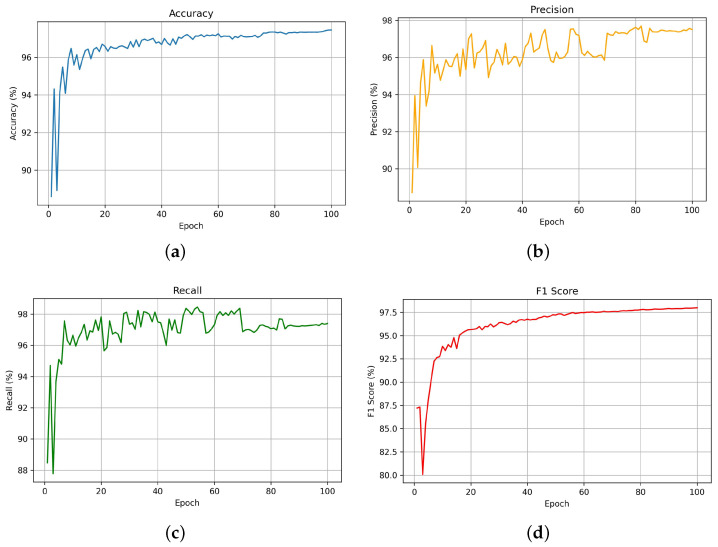
YOLOv11 model performance metrics. (**a**): Accuracy over training epochs. (**b**): Precision over training epochs. (**c**): Recall over training epochs. (**d**): F1-score over epochs.

Figure 5 illustrates the performance of the YOLOv11 model over 100 epochs across four key evaluation metrics, including accuracy, precision, recall, and the F1-score. The model demonstrates a consistent increase in accuracy, which stabilizes above 99% after 45 epochs, indicating high classification reliability. Figure 5b exhibits the precision metric, which determines the proportion of correct positive predictions among all positive predictions. The precision metric demonstrates progress toward 97.5% while exhibiting intermittent variations throughout the training process, which shows the model’s enhanced capability to reduce false positives. Figure 5c shows recall performance by measuring how well the model identifies all relevant cases without missing any (i.e., minimizing false negatives). The recall metric reaches above 96% and stays at that level from epoch 21 onwards, which shows that the model has attained effective detection sensitivity. Figure 5d displays the F1-score, which represents the harmonic mean of precision and recall. The F1-score metric shows rapid improvement and converges at approximately 97%, which proves that the model achieves a balance between precision and recall performance. YOLOv11 demonstrates robust stability and accuracy in recognizing the *Hand_flapping*, *Head_shaking*, *Rocking*, and *non_autistic* classes as shown by the performance curves.

### 6.2. Cross-Validation Results

We conducted a 5-fold cross-validation experiment with video-level partitioning to test the generalizability of our YOLOv11-based system. Table 2 presents the average values and standard deviations for performance metrics from each fold of the experiment. The results show exceptional consistency and minimal variability between splits, which proves the model’s robustness.

These results further support the stability of the model under different training–validation configurations and validate the reliability of the findings reported in the main evaluation.

### 6.3. Model Performance Comparison

**Figure 6:**
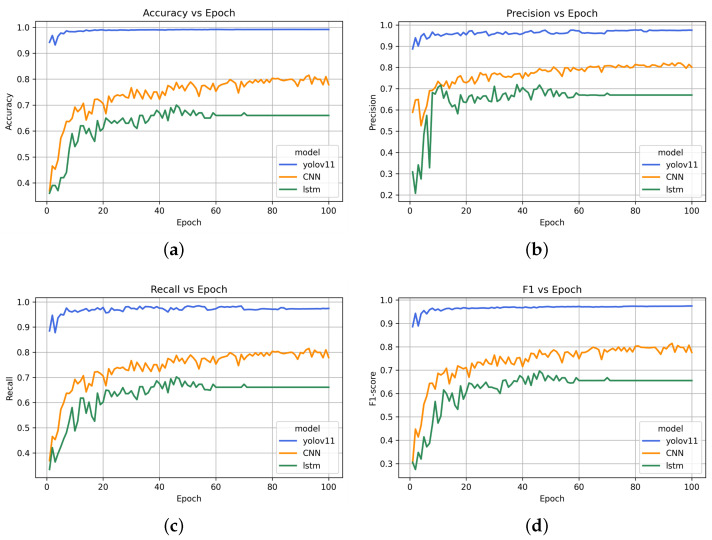
Model performance comparison. (**a**): Accuracy. (**b**): Precision. (**c**): Recall. (**d**): F1-score.

The comparative performance analysis of three deep learning models, including YOLOv11, the CNN (MobileNet-SSD), and LSTM, based on accuracy, precision, recall, and the F1-score is presented in Figure 6 after 100 epochs. The models received training to identify three primary behavioral indicators found in individuals with ASD, specifically hand flapping, body rocking, and head shaking. Figure 6a demonstrates that YOLOv11 achieves remarkable accuracy rates that surpass 95% at the beginning and later exceed 98%, but the CNN and LSTM models only reach about 78% and 65% accuracy, respectively. YOLOv11 demonstrates superior performance in both detecting and categorizing behavioral patterns related to ASD. YOLOv11 shows superior performance by keeping precision above 95%, whereas the CNN and LSTM stabilize near 80% and 67%, respectively, which shows YOLOv11’s higher efficiency in reducing false positives for ASD indicators. The performance comparison in Figure 6c indicates that YOLOv11 surpasses CNN’s and LSTM’s recall capabilities by identifying more than 95% of the actual ASD-related behaviors while the CNN and LSTM remain below at 78% and 65% recall rates. YOLOv11 displays enhanced sensitivity when it comes to recognizing subtle movement patterns, including repetitive body motions. Figure 6d depicts the F1-score, which serves as a balanced metric for precision and recall. YOLOv11 maintains F1-scores above 94%, whereas the CNN reaches its maximum near 78%, and LSTM performs the weakest among the models. This study’s results underscore YOLOv11’s exceptional strength and adaptability in identifying essential atypical movement patterns for early ASD analysis while establishing its potential as a viable option for live video behavioral screening applications.

The overall performance of the YOLOv11, CNN (MobileNet-SSD), and LSTM models based on key metrics (i.e., accuracy, precision, recall, and the F1-score) is presented in Table 3. YOLOv11 achieves superior results compared to other models with 0.99 accuracy, 0.96 precision, and 0.97 in recall and the F1-score. The model demonstrates strong detection capabilities for ASD-related behaviors while maintaining low rates of false positives and false negatives. The CNN achieves a balanced yet lower performance level across all metrics with a score of 0.87, which shows only moderate generalization, but it falls short in sensitivity and precision for detailed movement detection. The LSTM model produces the least results by reaching convergence with accuracy at 0.70 and an F1-score of 0.69, which demonstrates that temporal modeling by itself falls back in accurately recognizing ASD behaviors from video data. The findings demonstrate how object detection-based models such as YOLOv11 outperform others in delivering precise behavioral analysis.

The experimental data supports that the YOLOv11 model demonstrates outstanding performance in detecting ASD behavioral indicators such as hand flapping, body rocking, and head shaking from video-based datasets. YOLOv11 demonstrated superior performance compared to both the CNN (MobileNet-SSD) and LSTM models in every measured metric, including accuracy, precision, recall, and the F1-score, during extensive evaluations with multiple comparative baselines. The findings demonstrate that object detection-based architectures succeed in recognizing subtle repetitive ASD movements while maintaining minimal false detections in real-time applications. YOLOv11 demonstrates significant potential for implementation in assistive diagnostic tools and educational settings, as well as early intervention systems based on its high precision and recall rates. The model proves its strong robustness and generalization capabilities by achieving high performance on various ASD-related behavior categories during real-time operation, which confirms its suitability for deployment across different environments. This makes the model dependable for automated behavioral analysis systems designed to aid clinicians and researchers, as well as caregivers, in ASD screening and monitoring.

YOLOv11’s performance is superior to baseline approaches because it is a spatial detector combined with a class-specific attention mechanism. In YOLOv11, the C3k2 block is adopted, which is a basic component for all spatial dimensions of the network, enabling it to capture minute and localized movements such as flapping hands or head shaking. In addition, unlike a sequence model (e.g., LSTM), the YOLOv11 model is more appropriate for real-time behavioral screening because it processes frame-wise detection with high efficiency, offering both speed and accuracy.

YOLOv11 shows promising results on the gathered dataset, but there are some limitations that should be acknowledged. First of all, the size of the dataset is relatively small, and it does not represent well the variance in age and gender, as well as the environmental background. While the splits of the data were video-wise, the demographic distribution was not controlled, which can lead to potential hidden biases. Secondly, while data augmentation was performed, it is possible that the background features had an effect on ASD behavioral indicator detection due to the limited and possibly unrepresentative corpus. Last but not least, as the comparison of the proposed model with the baseline CNNs and LSTMs is limited by the size of the available dataset, larger, more diverse, and well-balanced datasets by the demographic parameters should be used in future works to test its applicability.

### 6.4. Computational Complexity Analysis

Our deployment feasibility assessment of the proposed system involved evaluating the computational complexity of the YOLOv11, CNN (MobileNet-SSD), and LSTM models by measuring the inference speed, model size, and parameter count through the NVIDIA Tesla V100 GPU.

As shown in Table 4, MobileNet-SSD delivered the quickest inference rate of 72 frames per second (FPS), together with a minimal model size of 17 MB, which makes it ideal for edge devices that operate under severe memory limitations. Performance issues in detection accuracy emerged alongside limited recall capabilities for nuanced ASD behaviors. YOLOv11 delivered real-time detection at 60 FPS while demonstrating better accuracy thanks to its enhanced attention modules and improved feature extraction capabilities despite being slightly larger in size. The LSTM model demonstrated temporal sequence modeling capabilities but performed with the greatest computational expense because of its sequential processing demands, which led to higher memory consumption and an average frame rate of only 28 FPS.

YOLOv11 demonstrates outstanding detection performance and computational efficiency, which makes it a prime option for scalable, real-time ASD behavior recognition applications in clinical and home settings.

### 6.5. Traditional Feature-Based Baselines and Comparison

In Table 5, we quantitatively compared our YOLOv11 model with the performance of other traditional, feature-based approaches. Although Local Binary Patterns (LBPs), a Histogram of Oriented Gradients (HOG), and a Local Binary Pattern on Three Orthogonal Planes (LBP-TOP) combined with a Support Vector Machine (SVM) classifier showed an acceptable performance, they are quite behind in all evaluated criteria compared to YOLOv11. These outcomes highlight the benefits of using deep learning-based object detection in the fine-grained motion analysis necessary for the ASD-related behavior recognition task, especially when processing subtle and repetitive patterns that are hard to encode with handcrafted features.

YOLOv11 outperforms traditional feature-based methods in ASD-typical behavior detection due to its end-to-end trainable architecture, which learns complex spatial hierarchies and contextual motion patterns from raw input data, enabling superior generalization capabilities. Traditional methods such as Local Binary Patterns (LBPs), a Histogram of Oriented Gradients (HOG), and Local Binary Patterns from Three Orthogonal Planes (LBP-TOPs) are typically limited by their handcrafted nature, offering less expressiveness in capturing the subtle and dynamic patterns of repetitive behaviors associated with ASD, especially in uncontrolled, real-world settings. These models are also less adept at handling occlusions, pose variations, and cluttered backgrounds compared to deep learning approaches like YOLOv11, which leverage multi-scale feature aggregation and spatial attention mechanisms (e.g., C3k2, SPPF, and C2PSA) to focus on and classify fine-grained motion cues across different lighting conditions and viewpoints. Furthermore, YOLOv11’s single-shot detection architecture provides real-time processing capabilities and the ability to perform simultaneous localization and classification, which is highly advantageous for live screening scenarios where both speed and accuracy are paramount.

## 7. Conclusions and Future Work

This research paper introduces an innovative ASD-typical behavior detection system based on body movement analysis through the YOLOv11 deep learning model, which identifies and categorizes behaviors including hand flapping, body rocking, and head shaking. The system utilizes a modular design that integrates the monitoring, network, cloud, and ASD-typical behavior detection layers to support real-time video analysis and behavior classification within dynamic environments. The combination of YOLOv11 with specific components such as C3k2, SPPF, and C2PSA leads to enhanced spatial–temporal understanding, delivering better accuracy, precision, recall, and F1-score results compared to CNN (MobileNet-SSD) and LSTM baselines. The combination of YOLOv11 in a modular design creates the first real-time ASD screening tool (i.e., to the best of our knowledge), which uses object detection to identify body movement indicators. The system provides an applicable solution for both home and clinical use while showcasing technical feasibility. In addition, we have gathered our own dataset comprising 72 videos, yielding a total of 13,640 images, which consist of four different behavior categories and have been annotated from public sources with validation by certified autism specialists. The dataset demonstrated strong generalization and sensitivity capabilities suitable for ASD-typical behavioral screening applications. This system represents an essential advancement toward automatically detecting ASD through non-invasive methods by analyzing observable behaviors while paving the way for intelligent diagnostic tool development.

The system demonstrated promising outcomes, but existing limitations indicate areas for further research. We acknowledge the limited data availability for baseline CNN and LSTM comparisons and suggest future studies with larger, demographically balanced datasets to assess model generalizability. Our current dataset documents common repetitive behaviors, but further research could incorporate additional ASD indicators like gaze aversion and echolalia to provide a wider behavioral profile. The system currently uses RGB video inputs, but future versions could use depth sensors or wearable inertial measurement units (IMUs) for better motion tracking in cluttered areas or dark settings. Subsequent research should extend beyond initial testing through prolonged use in residential and medical settings while involving caregivers and specialists in studies that assess the system’s usability and dependability, as well as its effect on early diagnosis processes.

## Figures and Tables

**Table 1:** Model training hyperparameters and configurations.

Parameter	CNN (MobileNet-SSD)	YOLOv11	LSTM
Epochs	100	100	100
Optimizer	Adam	SGD with momentum	Adam
Learning Rate	0.001	0.01 (cosine decay)	0.0005
Batch Size	32	32	64
Loss Function	Cross-entropy	Composite (GIoU, obj, and cls)	BCE + MSE (temporal window)
Input Size	224 × 224	640 × 640	640 × 640
Sequence Length	N/A	N/A	16-frame windows
Temporal Context	N/A	Frame-wise	Sequence modeling

**Table 2:** YOLOv11 cross-validation results (mean ± Std over 5 folds).

Metric	Accuracy	Precision	Recall	F1-Score
YOLOv11	0.988 ± 0.004	0.961 ± 0.006	0.966 ± 0.005	0.964 ± 0.005

**Table 3:** Overall performance comparison of determined models.

Model	Accuracy	Precision	Recall	F1-Score
YOLOv11	0.99	0.96	0.97	0.97
CNN	0.87	0.87	0.87	0.87
LSTM	0.70	0.71	0.70	0.69

**Table 4:** Computational performance comparison.

Model	Model Size (MB)	Parameters (M)	Inference Speed (FPS)
YOLOv11	38	12.4	60
CNN (MobileNet-SSD)	17	4.3	72
LSTM	92	22.1	28

**Table 5:** Performance comparison of deep learning and traditional feature-based models.

Model	Accuracy	Precision	Recall	F1-Score
YOLOv11	0.99	0.96	0.97	0.97
LBP + SVM	0.73	0.70	0.68	0.69
HOG + SVM	0.76	0.74	0.72	0.73
LBP-TOP + SVM	0.77	0.76	0.74	0.75

## Data Availability

A sample of the data used in this study (ASDBM_Dataset) is publicly available at (https://doi.org/10.34740/kaggle/dsv/12124307, accessed on 1 May 2025). The complete dataset can be obtained by contacting either the first author or the corresponding author.
